# Fatal 5-Fluorouracil Toxicity With Hyperammonemic Encephalopathy, Lactic Acidosis, and Pneumatosis Intestinalis Despite Uridine Triacetate Rescue: A Case Report

**DOI:** 10.7759/cureus.111669

**Published:** 2026-06-28

**Authors:** Hadi Matar, Erik Sembroski, Blake Buzard, Justin Shewmaker, Joseph R Blunck

**Affiliations:** 1 Department of Critical Care Medicine, Saint Luke's Hospital, Kansas City, USA; 2 School of Medicine, Saba University School of Medicine, The Bottom, NLD; 3 Department of Pharmacy, Saint Luke's Hospital, Kansas City, USA

**Keywords:** 5-fluorouracil, dpyd, fluorocitrate, fluoropyrimidine toxicity, hyperammonemia, lactic acidosis, pneumatosis intestinalis, uridine triacetate

## Abstract

5-Fluorouracil (5-FU) is among the most widely prescribed chemotherapeutic agents worldwide, yet severe toxicity can produce a life-threatening metabolic syndrome, most commonly associated with dihydropyrimidine dehydrogenase (DPD) deficiency. We report a fatal case of early-onset fluoropyrimidine toxicity characterized by severe metabolic derangements followed by severe myelosuppression.

A 56-year-old male patient with stage III (primary tumor (T4), regional lymph nodes (cN2), distant metastasis (cM0), human papillomavirus (HPV)-16-positive) squamous cell carcinoma of the right tonsil developed intractable vomiting within 24 hours of initiating continuous 5-FU infusion as part of a TPF regimen (docetaxel, cisplatin, 5-FU). He presented with severe lactic acidosis (lactate 14.8 mmol/L, rising to >17), hyperammonemia (ammonia 462 µmol/L), high anion gap metabolic acidosis (anion gap 30 mmol/L), and acute kidney injury. DPYD genotyping was ordered on the day of treatment, but the results were not available during hospitalization. The 5-FU infusion was discontinued before the completion of the planned four-day course. The patient was intubated for airway protection and transferred to a tertiary care center, and uridine triacetate was initiated approximately 24 hours after the discontinuation of 5-FU. Over 48 hours, the metabolic derangements improved (lactate 4.2 mmol/L, ammonia <10 µmol/L), though acute kidney injury continued to worsen. Subsequent myelosuppression developed by hospital day 3, with profound cytopenia (absolute neutrophil count (ANC) 0.02 × 10³/µL, platelets 26 × 10³/µL). The patient developed *Escherichia coli* bacteremia, vasopressor-dependent shock, and CT findings of extensive pneumatosis intestinalis with free intraperitoneal air. Surgical consultation estimated an 80% complication risk and 70% mortality for exploratory laparotomy. The family elected comfort care, and the patient died shortly after. Three weeks later, DPYD testing returned negative for all 12 variants analyzed (activity score 2.0, normal metabolizer).

This case documents severe fluoropyrimidine toxicity presenting with hyperammonemic encephalopathy, lactic acidosis, and myelosuppression, findings biologically consistent with fluorocitrate-mediated mitochondrial dysfunction supported by preclinical evidence. Resolution of the initial metabolic crisis should not be interpreted as resolution of overall toxicity, as the myelosuppressive component must be anticipated. DPYD genotyping results should be available prior to the initiation of fluoropyrimidines. Genotyping in this patient returned negative for all tested variants, demonstrating that fatal fluoropyrimidine toxicity can occur in patients classified as normal metabolizers by current testing panels. The predisposing factor underlying this patient's sensitivity to 5-FU remains unexplained.

## Introduction

Fluoropyrimidines such as 5-fluorouracil (5-FU) and its oral prodrug capecitabine remain among the most widely prescribed chemotherapeutic agents worldwide, used in treatment regimens for colorectal, head and neck, gastric, breast, and pancreatic malignancies. Despite decades of clinical use, severe toxicity is still a major treatment-limiting complication and can rarely be fatal [1].

​The primary determinant of 5-FU toxicity is dihydropyrimidine dehydrogenase (DPD), the rate-limiting enzyme that catabolizes approximately 80% of the administered 5-FU. Partial or complete DPD deficiency, most commonly caused by pathogenic variants in the DPYD gene, results in prolonged 5-FU exposure and toxic metabolite accumulation. Carriers of pathogenic DPYD variants are at substantially increased risk of fluoropyrimidine toxicity and treatment-related mortality [2,3]. Multiple DPYD variants have been identified as clinically actionable, and consensus guidelines note that testing panels limited to a subset of variants have lower sensitivity [4].

​Prospective studies show that DPYD genotype-guided dosing reduces fluoropyrimidine toxicity without impairing efficacy. However, implementation in the United States is still inconsistent [2]. The FDA issued a safety communication in 2024, updating the 5-FU label to include information on DPD deficiency, recognizing its importance [5].

A plausible mechanism underlying severe 5-FU toxicity is fluorocitrate-mediated inhibition of the tricarboxylic acid (TCA) cycle, which may contribute to simultaneous lactic acidosis and hyperammonemia. This has been characterized in preclinical models, but few reports describe serial metabolic trends [6-8]. We present a case of fatal 5-FU toxicity in which laboratory monitoring tracked the progression of metabolic toxicity consistent with prior mechanistic models of fluorocitrate-mediated toxicity, despite timely uridine triacetate administration. This case illustrates a plausible biochemical mechanism of toxicity and raises questions about the restrictions of current DPYD testing panels in predicting severe fluoropyrimidine toxicity.

## Case presentation

Patient information and history

A 56-year-old male patient (height 1.78 m, weight 93 kg) was diagnosed with squamous cell carcinoma of the right tonsil after presenting with right-sided jaw pain and dysphagia. Staging workup established the diagnosis as stage III (primary tumor (T4), regional lymph nodes (cN2), distant metastasis (cM0), human papillomavirus (HPV)-16 positive) with bilateral cervical nodal metastases. The treatment plan consisted of two cycles of induction TPF chemotherapy (docetaxel 142 mg (65 mg/m²), cisplatin 175 mg (80 mg/m²), and 5-FU 8,760 mg (1,000 mg/m²/day × four days as continuous infusion)) every 21 days, followed by definitive radiation therapy. The patient received docetaxel and cisplatin as outpatient infusions and was discharged the same day with a portable continuous 5-FU infusion pump to complete the first TPF cycle. DPYD genotyping samples were collected on the day chemotherapy was initiated.

Hospital Day 0: Emergency Department (ED) Presentation and Initial Management

Within 24 hours of initiating continuous 5-FU infusion, the patient developed intractable vomiting, malaise, weakness, and watery diarrhea and presented to an outside ED. On examination, he was fatigued and ill-appearing with dry mucous membranes. Vital signs showed the following: blood pressure 158/105, heart rate 89, respiratory rate 15, temperature 97.5°F, and oxygen saturation 98% on room air. Initial laboratories revealed high anion gap metabolic acidosis, lactic acidosis (12.2 mmol/L), acute kidney injury, and hyperuricemia (refer to Table 1). Arterial blood gas (ABG) on room air showed a mixed primary respiratory alkalosis with hyperventilation beyond the expected compensatory response to the primary metabolic acidosis (refer to Table 1). Complete blood count (CBC), liver function tests (LFTs), and procalcitonin were unremarkable. The drug screen was negative.

**Table 1:** Serial laboratory values at key inflection points during the hospital course pCO2: partial pressure of carbon dioxide; HCO3: bicarbonate; WBC: white blood cell; ANC: absolute neutrophil count; 5-FU: 5-fluorouracil; ABX: antibiotics

Laboratory parameter	Reference range	Day 0 (presentation)	Day 1 (AM)	Day 1 (PM)	Day 2 (PM)	Day 3 (AM)	Day 3 (PM)
Lactate (mmol/L)	0.5-2.2	12.2 → 14.8 → 20.8	>17	9	5	4.2	4
Ammonia (µmol/L)	15-45	386	462	159	124	24	10
pH (arterial)	7.35-7.45	7.36	7.51	7.55	7.48	7.41	7.28
pCO2 (mmHg)	35-45	21	19	26	27	30	39
HCO3 (mEq/L)	22-26	12	15.2 → 23.8	22.7	20.1	19	17.3
Anion gap (mmol/L)	8-12	29 → 30	26	21	18	17	17
Creatinine (mg/dL)	0.7-1.3	1.5	1.68	2	2.42	2.75	3
WBC (×10³/µL)	4.5-11	8	10.38	-	21.69	1.20 → 1.03	0.95
ANC (×10³/µL)	1.5-8	6	9.37	-	-	0.14	0.02
Platelets (×10³/µL)	150-400	456	384	-	201 → 61	51	26
Key clinical events	-	Vomiting, 5-FU discontinued, lactulose started	Intubated, ICU admit, bicarbonate infusion	Uridine triacetate started	Norepinephrine started, bicarbonate discontinued, rifaximin added	Cytopenias, *Escherichia coli* bacteremia, empiric ABX	Pneumatosis intestinalis on CT, vasopressor support, comfort care

The 5-FU infusion pump was disconnected. Two liters of normal saline were administered without improvement; lactate rose to 14.8 mmol/L. A single empiric dose of piperacillin-tazobactam and vancomycin was administered given the severity of illness, though clinical suspicion for infection was low and antibiotics were not continued. CT of the head was unremarkable. CT of the abdomen and pelvis showed no evidence of an acute process. Ammonia returned critically elevated at 386 µmol/L. Lactulose was initiated.

Over the next several hours, the patient's condition deteriorated rapidly. Lactate rose to 20.8 mmol/L, ammonia rose to 458 µmol/L, and bicarbonate fell to 6 mEq/L. The patient became profoundly encephalopathic with severe agitation and restlessness despite the initiation of dexmedetomidine and lorazepam sedation. The onset of lactic acidosis, hyperammonemia, and high anion gap metabolic acidosis shortly after 5-FU initiation raised the clinical suspicion for 5-FU toxicity. He was intubated for airway protection prior to air transport to a facility capable of uridine triacetate administration and providing renal replacement therapy if needed.

Hospital Day 1: Transfer With ICU Admission

On ICU arrival overnight, the patient was intubated and not following commands, with flexion withdrawal to painful stimulation in all extremities and sluggishly reactive pupils. Initial laboratories confirmed persistent severe metabolic derangement: ammonia 462 µmol/L, lactate >17 mmol/L, acute kidney injury, and hyperuricemia. ABG showed a pH of 7.51 and a partial pressure of carbon dioxide (pCO2) of 19 on a fraction of inspired oxygen (FiO2) of 40%, indicating mixed respiratory alkalosis and metabolic acidosis. Sodium bicarbonate infusion was started. Eight hours later, metabolic parameters began to improve. Lactate decreased from >17 to 9 mmol/L, ammonia from 462 to 159 µmol/L, and anion gap from 26 to 21 mmol/L. Repeat ABGs showed pH of 7.55-7.57 with pCO2 of 25-26 mmHg. Bicarbonate had improved to 23.8 mEq/L, and the infusion was discontinued.

Oncology initiated uridine triacetate 10 g via percutaneous endoscopic gastrostomy (PEG) every six hours and filgrastim 480 mcg subcutaneously daily. Rasburicase IV 3 mg was administered for hyperuricemia with concern for tumor lysis syndrome. Nephrology recommended supportive care with no indication for renal replacement therapy at this time. MRI of the brain revealed a T2-weighted/fluid-attenuated inversion recovery (T2/FLAIR) hyperintense signal within the bilateral thalami (left greater than right) (Figure 1).

**Figure 1:**
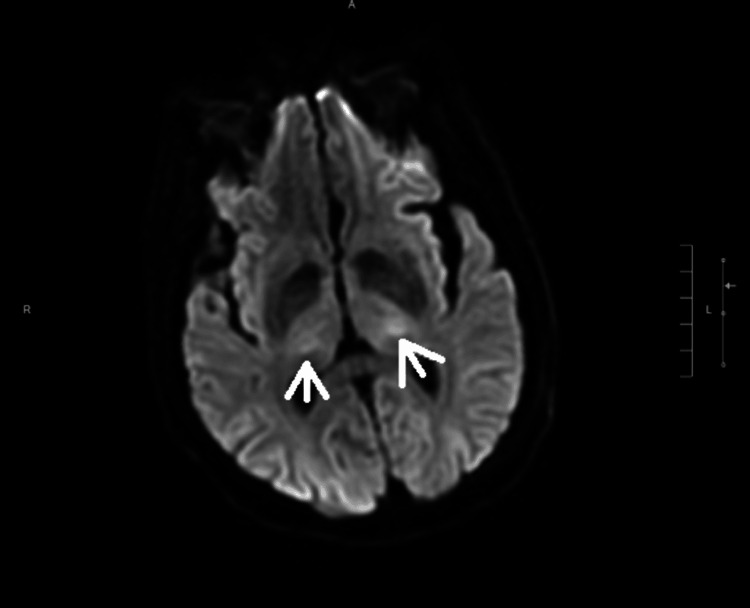
MRI of the brain with and without contrast (hospital day 1): axial T2/FLAIR images demonstrating bilateral thalamic hyperintensities Axial T2/FLAIR sequences demonstrate hyperintense signal involving the bilateral thalami (left greater than right), posterior midbrain, pons, and medulla, consistent with toxic-metabolic encephalopathy secondary to hyperammonemia. T2/FLAIR: T2-weighted/fluid-attenuated inversion recovery

Throughout the day, the patient remained critically ill in the ICU, intubated and mechanically ventilated, with toxic-metabolic encephalopathy in the setting of recent hyperammonemia. He was receiving antidotal and supportive therapy with close monitoring.

Hospital Day 2: Stabilization

Early on day 2, the patient remained persistently tachypneic, overbreathing the ventilator at excessive rates and tidal volumes despite escalating sedation. After further increases in fentanyl (150 mcg/hr) and propofol (80 mcg/kg/min), the respiratory rate improved, and pH trended down to 7.48.

The patient developed acute hypotension following a large bowel movement, possibly reflecting vagal response versus early bowel compromise. A fluid bolus and phenylephrine 100 mcg IV provided only transient improvement, and norepinephrine was initiated for sustained hemodynamic support. Metabolic parameters continued to improve throughout the day: lactate decreased from 9.6 to 5.0 mmol/L, ammonia decreased from 163 to 124 µmol/L, and the anion gap decreased from 20 to 18 mmol/L. Creatinine rose further to 2.42 mg/dL (estimated glomerular filtration rate (eGFR) 30.6 mL/min/1.73 m²). Uric acid normalized to 3.0 mg/dL after rasburicase. Rifaximin was added for hyperammonemia management. Uridine triacetate was continued, and enteral nutrition was initiated. Blood cultures drawn at the outside ED remained without growth at 48 hours.

Hospital Day 3: Deterioration

Overnight, ammonia normalized to 24 µmol/L and lactate stabilized at 4.0-4.2 mmol/L. ABG showed a pH of 7.41, pCO2 30 mmHg, and bicarbonate 19 mEq/L, the first normal pH since admission. White blood cell (WBC) count increased overnight to 21.69 × 10³/µL and then dropped to 1.2 × 10³/µL. Platelet levels decreased from 201 to 61 × 10³/µL, while hemoglobin remained stable. Lactulose was held due to high-volume stool output (2,000 mL via rectal tube), and tube feeds were held as the abdomen was now distended. Low-grade fevers developed (Tmax 100.7°F). That morning, an abdominal X-ray showed gaseous distention of the colon and small bowel. Linezolid 600 mg IV q12h and piperacillin-tazobactam 3.375 g IV q6h were initiated with concern for neutropenic fever.

By the afternoon, CBC continued to worsen: WBC 1.03 × 10³/µL, ANC 0.14 × 10³/µL, platelets 51 × 10³/µL, and hemoglobin 13.0 g/dL. Vasopressor requirements escalated, and vasopressin was added. The abdomen became increasingly firm and distended. Blood cultures turned positive for *Escherichia coli* via polymerase chain reaction (PCR). Repeat CBC showed the following: WBC 0.95 × 10³/µL, ANC 0.02 × 10³/µL, and platelets 26 × 10³/µL. Creatinine rose to 3.13 mg/dL (eGFR 22.5 mL/min/1.73 m²). Ionized calcium was critically low at 3.2 mg/dL and was replaced. ABG showed progressive deterioration, with pH falling from 7.41 to 7.28 over the day with a rising base deficit despite stable pCO2, indicating worsening metabolic acidosis. Lactate began to rise again (3.2 → 4.1). Ammonia remained suppressed.

CT of the abdomen and pelvis revealed extensive bowel distention with pneumatosis intestinalis and tiny bubbles of free intraperitoneal air with ascites, suspicious for bowel necrosis (Figure 2). General surgery was consulted emergently and calculated a National Surgical Quality Improvement Program (NSQIP) risk estimate of 80% for complications and 70% mortality for an exploratory laparotomy. The patient's family, noting the surgical risk and his previously expressed wishes to be do not resuscitate/do not intubate (DNR/DNI) (temporarily reversed at the time of intubation to facilitate transfer), elected to transition to comfort care. The patient was palliatively extubated with family at bedside, and death occurred shortly after.

**Figure 2:**
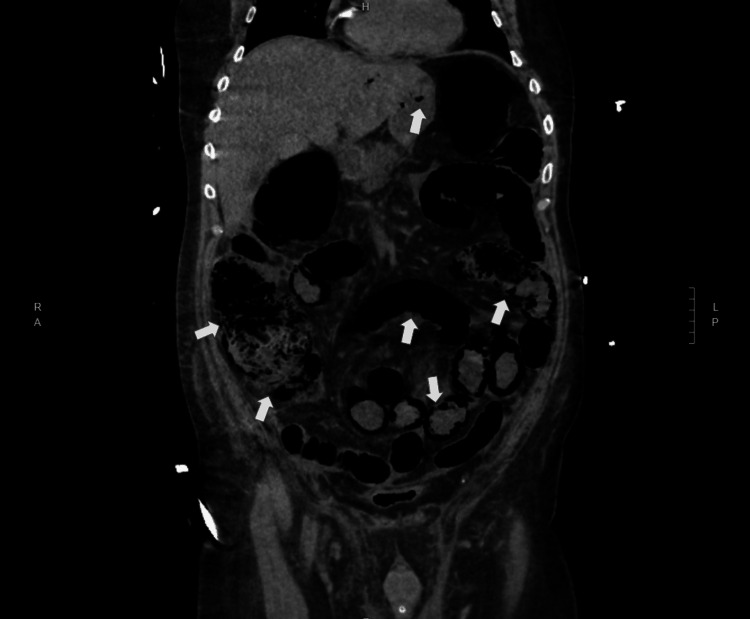
CT of the abdomen and pelvis without contrast (hospital day 3) demonstrating pneumatosis intestinalis and pneumoperitoneum Coronal images demonstrating extensive bowel distention with pneumatosis intestinalis (intramural gas) and tiny bubbles of free intraperitoneal air, suspicious for bowel necrosis with impending perforation.

## Discussion

This case portrays early metabolic toxicity shortly after 5-FU initiation, followed by cytopenia, bacteremia, and bowel necrosis despite antidotal therapy. The clinical course raises several important considerations pertaining to the metabolic manifestations of fluoropyrimidine toxicity, hematologic complications, and the limitations of uridine triacetate rescue.

The fluorocitrate mechanism

The primary antineoplastic mechanism of 5-FU is the inhibition of thymidylate synthase via its active metabolite, fluorodeoxyuridine monophosphate (FdUMP), disrupting DNA synthesis [9]. The metabolic toxicity observed in this case appears to arise from a separate catabolic pathway. The severe lactic acidosis, hyperammonemia, and high anion gap metabolic acidosis are biologically consistent with potential fluorocitrate-associated inhibition of aconitase in the TCA cycle (Figure 3 and Figure 4). The temporal association and metabolic profile suggest a fluoropyrimidine-related toxicity syndrome; however, the proposed mechanistic interpretation is based on the integration of preclinical data.

**Figure 3:**
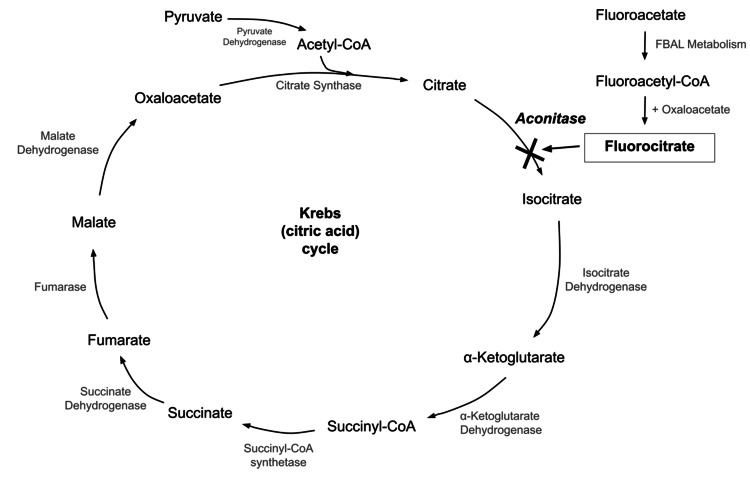
The Krebs (citric acid) cycle demonstrating the proposed inhibition of aconitase by fluorocitrate, a toxic metabolite of 5-FU The arrow with an X indicates the blocked citrate-to-isocitrate step. 5-FU: 5-fluorouracil; FBAL: α-fluoro-β-alanine Original figure created by author H.M. using presentation software (Microsoft PowerPoint, Microsoft Corp., Redmond, WA, USA), based on Arellano et al. [6], Nishikawa et al. [8], and Boilève et al. [10].

**Figure 4:**
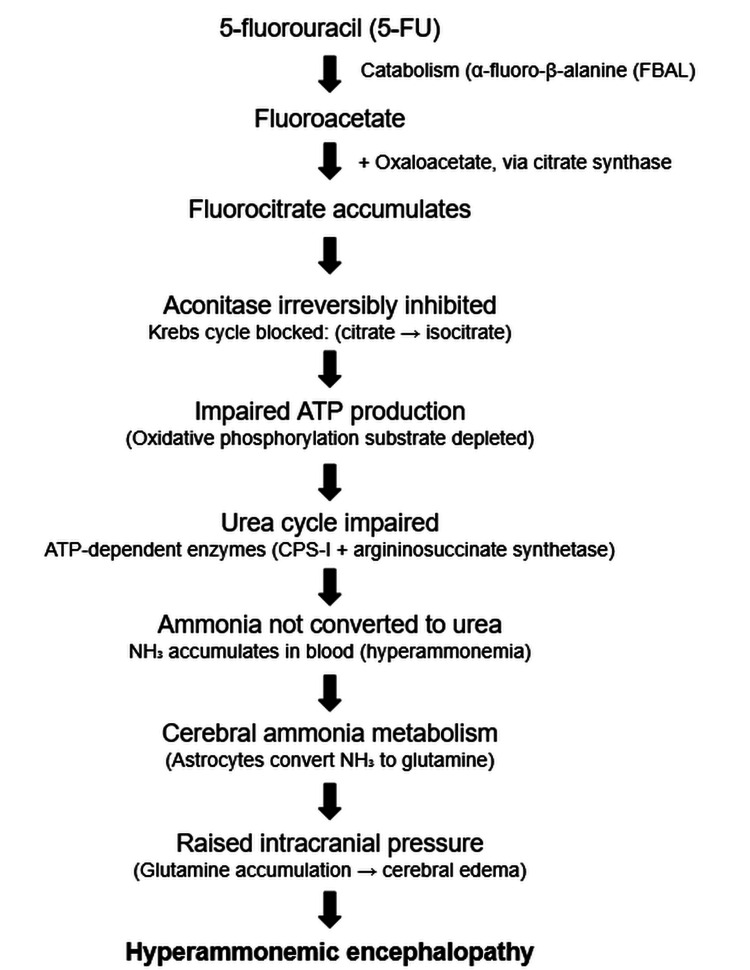
Proposed mechanism of 5-FU-induced hyperammonemic encephalopathy 5-FU: 5-fluorouracil; ATP: adenosine triphosphate; CPS-I: carbamoyl phosphate synthetase I Original figure created by author H.M. using presentation software (Microsoft PowerPoint, Microsoft Corp., Redmond, WA, USA), based on Arellano et al. [6], Nishikawa et al. [8], and Boilève et al. [10].

The major 5-FU catabolite α-fluoro-β-alanine (FBAL) undergoes additional metabolism to fluoroacetate, first demonstrated as a 5-FU metabolite by Arellano et al. [6]. Fluoroacetate is condensed with oxaloacetate by citrate synthase to form fluorocitrate, which potently inhibits aconitase, the enzyme converting citrate to isocitrate [6-8]. Nishikawa et al. directly measured elevated levels of FBAL and fluoroacetate in a patient with 5-FU-associated hyperammonemia [8], supporting the proposed pathways (Figure 3 and Figure 4).

Aconitase inhibition produces two downstream effects. First, disruption of the TCA cycle may impair aerobic adenosine triphosphate (ATP) production, shifting cellular metabolism toward anaerobic glycolysis and thereby generating type B lactic acidosis [6-8]. Second, the urea cycle, which is highly ATP-dependent at the carbamoyl phosphate synthetase I step, may fail as cellular ATP stores become depleted, leading to ammonia accumulation [8,10]. TCA cycle blockade also depletes α-ketoglutarate, which is required for transamination reactions that channel nitrogen into the urea cycle. In our case, both lactate and ammonia improved in parallel over 48 hours, consistent with a common upstream metabolic disturbance.

Myelosuppression

The most clinically relevant finding in this case is the dissociation between metabolic and hematologic toxicity. By hospital day 3, the patient appeared to be improving: lactate 4.2 mmol/L, ammonia <10 µmol/L, and anion gap 16 mmol/L. However, hematologic toxicity subsequently emerged. The ANC fell to 0.02 × 10³/µL and the platelet count to 26 × 10³/µL. This nadir occurred approximately 4-5 days post-chemotherapy, well before the expected 9-14-day nadir for 5-FU [11]. However, docetaxel and cisplatin may have also contributed to the severity of myelosuppression.

The severe neutropenia likely contributed to enterocolitis, which was followed by pneumatosis intestinalis and *Escherichia coli* bacteremia. Neutropenic enterocolitis results from chemotherapy-induced mucosal injury followed by bacterial invasion of the bowel wall in the absence of immune defense. A systematic review of 227 cases demonstrated that neutropenic enterocolitis carries significant mortality, particularly when associated with sepsis and multiorgan failure [12].

In this patient, improvement in metabolic parameters preceded severe marrow suppression. Clinicians must anticipate the myelosuppression and prepare for its consequences. In this case, filgrastim was appropriately initiated on day 1.

Uridine triacetate

Uridine triacetate was FDA-approved in 2015 for the emergency treatment of patients exhibiting early-onset severe fluoropyrimidine toxicity within 96 hours of administration [13-15]. Uridine triacetate increases circulating uridine concentrations, therefore reducing the incorporation of toxic fluorouridine metabolites into RNA and partially rescuing normal cellular metabolism [13-15]. In the FDA review of uridine triacetate, 96% of patients met the major efficacy endpoint, defined as survival at 30 days or survival until resumption of chemotherapy, if prior to 30 days [13].

In this case, uridine triacetate was initiated approximately 24 hours after the discontinuation of 5-FU, well within the FDA-approved 96-hour window following the end of fluoropyrimidine administration. Following the discontinuation of 5-FU, uridine triacetate administration, lactulose therapy, and ongoing drug clearance, metabolic parameters improved substantially. Notably, uridine triacetate competes with fluorouridine metabolites at the RNA level. However, it does not directly reverse fluorocitrate-mediated TCA cycle inhibition, nor can it rescue hematologic injury caused by cytotoxic metabolites.

DPYD testing

DPYD genotyping was ordered on the day of chemotherapy initiation, but results were not available before treatment, during hospitalization, or at the time of death. Results returned three weeks later and were negative for all 12 variants tested (activity score 2.0, normal metabolizer). The panel included all seven Tier 1 variants and five of six Tier 2 variants recommended by the Association for Molecular Pathology/American College of Medical Genetics and Genomics/Clinical Pharmacogenetics Implementation Consortium (AMP/ACMG/CPIC) consensus, providing broader coverage than the typical three-to-four variant panel [4].

This result was notable given the severity and rapidity of toxicity. However, current DPYD testing has well-documented limitations in predicting severe fluoropyrimidine toxicity. The standard four-variant panel captures only an estimated 20-30% of patients who develop severe toxicity, and even expanded sequencing of 44 variants achieved a crude sensitivity of only 47.5% for grade 4-5 toxicity [16,17]. Meulendijks et al. found that DPD deficiency, as measured by enzyme activity testing, was identified in only 39-61% of patients experiencing severe fluoropyrimidine toxicity [2]. In a meta-analysis of 13,929 patients, Sharma et al. reported 14 treatment-related deaths among 13,363 patients without identified pathogenic DPYD variants [18].

These limitations illustrate the broader challenge of characterizing the risk of DPD deficiency. Approximately 1,600 DPYD sequence variants have been identified, most of which are rare and functionally uncharacterized [4]. In a cohort of over 10,000 individuals, sequencing identified 112 potentially significant variants in 630 individuals (6.3%) that would have been missed by a standard three-variant panel [4].

Taken together, these limitations raise the possibility that this patient harbored an undetected deleterious variant or a mechanism of DPD impairment not captured by current testing. The predisposing factor in this patient remains unexplained. Despite the negative result in this case, pre-treatment DPYD genotyping remains clinically important, as carriers of pathogenic variants have a 25.6-fold increased risk of treatment-related death [18]. The FDA Boxed Warning for fluorouracil recommends testing patients for genetic variants of DPYD prior to initiation unless immediate treatment is necessary [11].

Limitations

This case has several limitations. Although DPYD genotyping returned negative for all 12 variants tested, the panel does not capture the full spectrum of DPYD variants. Full DPYD gene sequencing and DPD phenotypic enzyme activity testing were not performed, so the hypothesis that this patient had an undetected rare variant or functional DPD deficiency is considered speculative. 5-FU serum levels and fluorocitrate metabolite measurements were not obtained. The contribution of cisplatin and docetaxel to the overall toxicity cannot be entirely excluded. Cisplatin in particular may have contributed to the nausea, vomiting, and acute kidney injury. Docetaxel and cisplatin may have contributed to the severity of myelosuppression. Alternative contributors to the metabolic abnormalities cannot be completely excluded, including sepsis-related hypoperfusion, bowel ischemia, aspiration-related hypoxia, pharmacological effects, and nutritional deficiency. However, the extremely early onset after 5-FU exposure, preserved hemodynamics at presentation, low procalcitonin, and parallel improvement in lactate and ammonia after drug discontinuation argue against these as the primary etiology. This is a single case, and the observations cannot definitively establish causation.

## Conclusions

This case documents severe fluoropyrimidine toxicity presenting with hyperammonemic encephalopathy, lactic acidosis, and subsequent myelosuppressive collapse, findings biologically consistent with fluorocitrate-mediated mitochondrial dysfunction supported by preclinical evidence. Despite the timely administration of uridine triacetate, the antidote could not reverse complications that had already occurred.

​Several clinical implications emerge. First, resolution of the initial metabolic crisis should not be interpreted as resolution of overall toxicity, as the delayed myelosuppressive component must be anticipated. Second, awareness of the proposed metabolic mechanism underlying both lactic acidosis and hyperammonemia may aid earlier recognition of fluoropyrimidine toxicity. Third, DPYD genotyping results should be available prior to fluoropyrimidine initiation, as recommended by the FDA Boxed Warning. While pre-treatment identification of a DPYD variant allows dose modification or drug avoidance, genotyping in this patient returned negative for all tested variants. This case demonstrates that fatal fluoropyrimidine toxicity can occur in patients classified as normal metabolizers by current testing panels and a negative result does not exclude the possibility of severe toxicity. The predisposing factor underlying this patient's extreme sensitivity to 5-FU remains unexplained.
